# Sulfonated Lignin-*g*-Styrene Polymer: Production and Characterization

**DOI:** 10.3390/polym10080928

**Published:** 2018-08-19

**Authors:** Nasim Ghavidel Darestani, Adrianna Tikka, Pedram Fatehi

**Affiliations:** Chemical Engineering and Chemistry Departments, Lakehead University, 955 Oliver Road, Thunder Bay, ON P7B 5E1, Canada; nghavide@lakeheadu.ca (N.G.D.); ahtikka@lakeheadu.ca (A.T.)

**Keywords:** polymerization, lignin, styrene, sulfonation, sustainable polymers, characterization, NMR

## Abstract

Among sustainable alternatives for replacing fossil-based chemicals, lignin is widely available on earth, albeit the least utilized component of biomass. In this work, lignin was polymerized with styrene in aqueous emulsion systems. The reaction afforded a yield of 20 wt % under the conditions of 100 g/L lignin concentration, pH 2.5, 0.35 mol/L sodium dodecyl sulfate concentration, 5 mol/mol styrene/lignin ratio, 5 wt % initiator, 90 °C, and 2 h. The lignin-*g*-styrene product under the selected conditions had a grafting degree of 31 mol % of styrene, which was determined by quantitative proton nuclear magnetic resonance (NMR). The solvent addition to the reaction mixture and deoxygenation did not improve the yield of the polymerization reaction. The produced lignin-*g*-styrene polymer was then sulfonated using concentrated sulfuric acid. By introducing sulfonate group on the lignin-*g*-styrene polymers, the solubility and anionic charge density of 92 wt % (in a 10 g/L solution) and −2.4 meq/g, respectively, were obtained. Fourier-transform infrared (FTIR), static light scattering, two-dimensional COSY NMR, elemental analyses, and differential scanning calorimetry (DSC) were also employed to characterize the properties of the lignin-*g*-styrene and sulfonate lignin-*g*-styrene products. Overall, sulfonated lignin-*g*-styrene polymer with a high anionicity and water solubility was produced.

## 1. Introduction

Lignin is the most abundant aromatic biomaterial in nature and has the potential to be used in various applications [1,2]. Currently, the commercial use of lignin is limited, as it is primarily used as an energy source. The chemical modification of lignin has gained attention in order to produce value-added products from lignin [3].

Recently, there have been noticeable reports on the production of water soluble polymers from lignin [4,5,6]. Researchers summarized different chemical modifications of lignin and its applications in reinforcement fillers, antioxidants, UV absorbents, antimicrobial agents, carbon precursors, biomaterials [7], gel-coated films [8], and composites [9,10].

Graft polymerization via free-radical polymerization enables the functionalization of lignin with different monomers, such as acrylates. Polymerization of lignin and cationic, anionic, amphoteric, and non-ionic monomers were studied in the past [11]. Fang and coworkers reported using acrylamide as an uncharged monomer in producing enzymatically hydrolyzed lignin-*g*-acrylamide polymers [12]. Kong and coworkers successfully polymerized acrylic acid (i.e., anionic monomer) and kraft lignin using free radical techniques and produced water-soluble lignin-*g*-acrylic acid polymers [13]. Lignin-*g*-acrylamide-*g*-[2-(methacryloyloxy) ethyl] trimethylammonium chloride copolymers (KL-*g*-AM-*g*-DMC) were also produced using free radical polymerization [5]. The functionalization was reported to impart valuable properties to lignin, making it more hydrophilic, hydrophobic or thermally stable [14,15].

In previous studies, polymers were produced using water soluble monomers. Styrene, on the other hand, is not water soluble, making its polymerization in aqueous based systems challenging. Styrene is a known monomer with several applications in nanocomposites [16], ion exchange resins, and adsorbent productions [17]. Reports are available on the polymerization of lignin and styrene following different techniques, such as atom transfer radical polymerization [18,19], radiation induced graft polymerization [20], or emulsion suspension polymerization [21]. Hilburg and coworkers produced lignin composites by polymerizing styrene and methyl methacrylate on lignin using atom transfer radical polymerization (ATRP) technique [19]. Wang and coworkers synthesized lignin graft copolymers via activator regenerated by electron transfer (ARGET) atom transfer radical polymerization (ATRP) [18]. Podkościelna and coworkers reported graft polymerization of styrene and divinylbenzene monomers on kraft lignin in a mixture of water and organic solvents (i.e., toluene and 1-decanol) in an emulsion polymerization system [21]. In three component systems, one monomer may be used as a crosslinker for joining lignin and the other monomers, improving the polymerization efficiency. The two component systems, however, require the reaction of lignin and another monomer in polymerization systems, which might be more challenging as lignin is generally not very reactive in polymerization reactions. In this regard, the polymerization of lignin and styrene in a two-component aqueous emulsion system using water soluble initiator has not been reported yet, which makes it the first objective of this work.

Sulfonation has been widely used as a means for generating sulfonated lignin products [22]. Sulfonated lignin has been used as dispersants and flocculants in the past [23]. The sulfonation of polystyrene (PS) has also been performed commercially [24]. Several approaches for sulfonating PS are available in the literature, such as using concentrated sulfuric acid in liquid form [25] or vapor form [26], combination of concentrated sulfuric acid with catalyst [27], chlorosulfonic acid [28], silica sulfuric acid [29], acetyl sulfate [30], and sulfur trioxide [31]. Sulfonated PS can be used as electrolyte fuel cells (PEFC) [32], capillary electrophoresis (CE) [33], flocculants, and cation-exchange resins [29]. If lignin-*g*-styrene is sulfonated, it may have similar applications to that of sulfonated lignin and/or PS. The second objective of this work was to investigate the sulfonation of lignin-*g*-styrene using concentrated sulfuric acid as sulfonating reagents [30].

The primary novelty of this study was the investigation on the production of lignin-*g*-styrene in an emulsion polymerization system and the sulfonation of the produced polymer. The reaction was optimized to obtain the highest yield in the emulsion polymerization reaction and the products were characterized using different techniques. The main aim of this study was the production of a high molecular weight and water-soluble lignin-based product via emulsion polymerization system.

## 2. Experimental

### 2.1. Materials

Softwood kraft lignin was received from a company located in south eastern USA (Domtar, Plymouth, NC, USA). Sodium dodecyl sulfate (SDS), dimethyl sulfoxide (DMSO), trimethylsilyl propanoic acid (TSP), dodecyl benzenesulfonic acid (DBAS), ethanol (80 vol %), potassium persulfate, sulfuric acid (98%), and acetone, all of analytical grade, were obtained from Sigma-Aldrich (Markham, Canada) and used as received without further purification. Styrene was also received from Sigma-Aldrich (Markham, ON, Canada). To remove inhibitors from styrene, it was first washed with NaOH (5%) solution and then with deionized water three times, each time followed by drying with anhydrous calcium chloride (CaCl_2_), then it was kept at 4 °C for further use [34]. Cellulose acetate dialysis membrane (with molecular weight cut off of 1000 g/mol) was obtained from Spectrum Laboratories, Inc., Rancho Dominguez, CA, USA. Polydiallyldimethylammonium chloride (PDADMAC) and potassium polyvinyl sulfate (PVSK) were obtained from Sigma-Aldrich (Markham, ON, Canada) and diluted to 0.005 M prior to use.

### 2.2. Polymerization of Lignin and Styrene 

The polymerization of lignin and styrene was conducted in an aqueous medium using an emulsion suspension polymerization methodology under various conditions. All reactions were performed in three neck flasks equipped with condensers. In these experiments, a 100 g/L lignin concentration was prepared in 30 mL deionized water and after mixing for 30 min, surfactant was added to the mixture and suspension was stirred for another 30 min. The pH of the suspension was adjusted to 2.5 using 20 wt % H_2_SO_4_ solution. Styrene and constant amount of initiator (5 wt %) were subsequently added. The reaction was initiated by transferring the three neck flask in an oil bath and stirring for 2 h at 90 °C. The polymerization reactions were repeated under different conditions of styrene/lignin molar ratios (0.5–20 mol/mol), surfactant types (sodium dodecyl sulfate (SDS) and dodecyl benzenesulfonic acid (DBAS)), concentrations (0.1–1.7 mol/L), reaction temperatures (70–95 °C), and reaction times (1–3 h). The impacts of solvent (DMSO) and nitrogen purging (i.e., oxygen-free environment) on the reaction medium were also investigated in this work.

After completion of the reaction, the mixtures were cooled to room temperature and the products of the reactions were precipitated from the systems by mixing the reaction media with ethanol. The precipitates were separated using centrifugation at 3500 rpm for 5 min, then they were dried in the oven at 60 °C for 2 days. Afterward, the products were purified in boiling acetone by Soxhlet extraction [35] and were then characterized. These products are denoted lignin-*g*-styrene (KL-*g*-PS) in this work. The yield of the polymerization reaction was determined following Equation (1) and the results are depicted in Table 1.

(1) $$Yield=\frac{W_{T}}{W_{L}+W_{s}}\times 100$$

where *W_T_* is the weight of KL-*g*-PS collected after Soxhlet extraction (*g*); and *W_L_* and *W_s_* are the weights (*g*) of lignin and styrene used in the polymerization reaction, respectively. The KL-*g*-PS products generated under different styrene/lignin molar ratios were collected for sulfonation experiments.

### 2.3. Sulfonation of Lignin-g-styrene

Sulfonation was performed with concentrated sulfuric acid in this work. Sulfonation was conducted on the KL-*g*-PS polymers produced under the conditions of 100 g/L lignin concentration, pH 2.5, 0.35 mol/L sodium dodecyl sulfate concentration, 5 wt % initiator, 90 °C, and 2 h reaction with different molar ratios of styrene/lignin (0.5, 1, 3, 5) in order to explore the effect of polystyrene chains in sulfonation feasibility. In this set of experiments, 1 g of KL-*g*-PS was transferred into three neck flasks. Sulfuric acid (98%) was added to the system at the ratio of 1/10 wt/v (KL-*g*-PS)/sulfuric acid. The reaction was carried out for 1 h at 90 °C. To stop the reactions, they were cooled to room temperature, and their pH was adjusted to 7 using 10 M NaOH. Ion impurities were separated from the sulfonated products (KL-*g*-SPS) via maintaining the products in dialysis membranes, while water was changed every 12 h for 2 days. The final products were dried at 105 °C in an oven overnight. The products of this step were named sulfonated lignin styrene polymer, KL-*g*-SPS.

KL-*g*-PS, which was produced under the reaction conditions of 100 g/L lignin concentration, pH 2.5, 0.35 mol/L sodium dodecyl sulfate concentration, 5 mol/mol styrene/lignin ratio, 5 wt % initiator, 90 °C, and 2 h, and its corresponding sulfonated polymer, KL-*g*-SPS, with the charge density of −2.4 mmol/g, were selected for the chemical and physical assessments.

### 2.4. Nuclear Magnetic Resonance (NMR) Analysis

The structures of unmodified lignin (KL) and KL-*g*-PS were analyzed by proton nuclear magnetic resonance spectroscopy of ^1^H and two-dimensional homonuclear correlation spectroscopy spectrum ^1^H–^1^H COSY. Trimethylsilyl propanoic acid (TSP) was used as internal standard for quantitative ^1^H-NMR analysis. KL and KL-*g*-PS were dissolved in 500 μL of DMSO-d_6_ to make 40–50 g/L concentrations, and 2 mg of TSP was also mixed with the solutions to generate reference peak in the NMR analysis [36]. The solutions were stirred at 100 rpm overnight prior to analysis. The analysis was performed using an INOVA-500 MHz instrument (Varian, Palo Alto, CA, USA). Adjustments for 1D ^1^H and 2D ^1^H–^1^H COSY are follows, respectively: 1D ^1^H: 64 number of scans, A 45° pulse width and a relaxation delay of 1.00 s, which was followed by 2D ^1^H–^1^H COSY: 4 scans per increment with 128 increments.

### 2.5. FTIR Analysis

The Fourier-transform infrared (FTIR) spectra of unmodified lignin, KL-*g*-PS and KL-*g*-SPS polymers were recorded in this set of experiments. The samples were dried in an oven prior to analysis, and 0.05 g of each sample was taken into account for analysis using an FTIR instrument (Bruker Tensor 37, Ettlingen, Germany, ATR accessory). The analysis was conducted in transmittance mode in the range of 500 and 4000 cm^−1^ with a 4 cm^−1^ resolution, and 32 scans per sample were conducted. 

### 2.6. Elemental Analysis

The organic elements of the samples were determined using an Elementar Vario EL Cube elemental analyzer (Langenselbold, Germany). After removing their moisture by putting samples in an oven at 105 °C overnight, 2 mg of KL, KL-*g*-PS, and KL-*g*-SPS were transferred in the integrated carousel of the elemental analyzer and burned at 1200 °C, which facilitated their carbon, hydrogen, nitrogen, and sulphur assessments.

### 2.7. Molecular Weight Determination

The weighted average molecular weights of KL, KL-*g*-PS, and KL-*g*-SPS were analyzed using a static light scattering, SLS, analyzer (BI-200SM Brookhaven Instruments Corp., Holtsville, NY, USA). The maximum solid-state laser power was 35 mW at the wavelength of 637 nm. The samples were dissolved in 20 mL of a 0.5 M NaOH with a 10 mM KNO_3_ by stirring at 300 rpm overnight at 25 °C to prepare various concentrations ranging from 0.2 to 2 mg/mL. Afterward, solutions were filtered using 0.45 μm syringe filters and transferred to 20 mL glass vials. The measured data was further analyzed using Zimm plot software (Holtsville, NY, USA) to obtain the absolute molecular weights. The results were reported based on three independent measurements. It was previously stated that lignin-*g*-polyacrylic acid with the molecular weight of 7 × 10^5^ g/mol had the hydrodynamic size of 35 nm [6]. Lignin-*g*-styrene polymer’s molecular weight was in the same range and thus they probably had a similar hydrodynamic size. Therefore, all of lignin-*g*-styrene probably passed through the filter. However, analysis on the impact of filtration on screening lignin-*g*-styrene polymer should be investigated in future.

### 2.8. DSC Analysis

The thermal stability of the samples was determined by a differential scanning calorimeter (TA instrument, Q2000 DSC, Brossard, QC, Canada) using the standard cell RC mode. The dried samples were analyzed in the heat/cool/heat mode between 30 and 250 °C at a rate of 5 °C/min and a 50 mL/min nitrogen flow. In the second heating cycle, the glass transition temperatures (*T*_g_) were recorded accordingly.

### 2.9. Charge Density Analysis

The charge density of produced KL-*g*-SPS samples was measured using a particle charge detector (PCD-04, Mutek, Eclépens, Switzerland). In this set of experiments, 1 mL of 10 g/L solution of KL-*g*-SPS was prepared and then titrated against PDADMAC standard solutions (0.005 M) as stated elsewhere [37].

## 3. Results and Discussion

### 3.1. Lignin-g-Styrene Polymerization

The polymerization reaction was conducted through a free radical emulsion polymerization system, which was initiated by potassium persulfate. The main components of emulsion systems were monomers, initiator, and surfactant in water. Lignin was the main component on which polymerization was initiated. By keeping the concentration of surfactant (SDS) above its critical micelle concentration, CMC (8.2 mM at 25 °C) [38], hydrophobic styrene monomers would diffuse into the micelles and form monomer micelles. In addition, styrene molecules could exist in monomer droplets, which would be formed by agglomeration of hydrophobic styrene molecules in water [39], and a small fraction of styrene molecules would dissolve in the aqueous system (0.03% at 20 °C) [40]. Lignin molecules, owing to their hydrophilic functional groups, would not be able to form micelles and were partially soluble in the aqueous medium. After formation of water soluble initiator radicals, polymerization would start with lignin macromolecules and presumably soluble styrene monomers in the system. As a result, when an essential chain length of polystyrene formed on lignin, the generated lignin based macroradicals would be sufficiently hydrophobic to enter the micelles [39]. The propagation would subsequently continue with styrene monomers in the micelles.

**Scheme 1:**
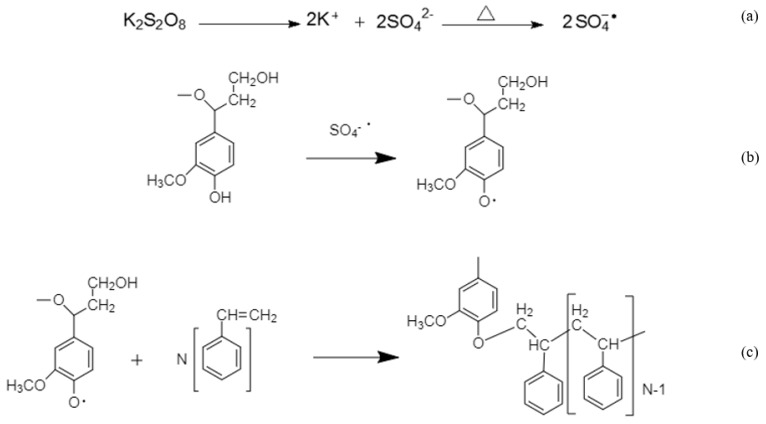
Lignin styrene polymerization.

The polymerization scheme of lignin and styrene is shown in Scheme 1. Sulfate radicals would be formed by heating the reaction medium (Scheme 1a), which would have the ability to release unstable hydrogen of phenol to form phenoxy radicals (Scheme 1b) [41]. Lignin had two possible positions on its backbone for the production of active radicals; one on its phenol group and the other one on its aliphatic hydroxyl group [42]. Kong et al. reported that the polymerization of lignin with acrylic acid was favorable on the phenol group because the phenol group could produce more stable radical than the aliphatic hydroxyl group [13]. Polymerization would be initiated following the attack of phenoxy radicals to the vinyl group of styrene (Scheme 1c) [41]. Continuing the polymerization, the π bond between two carbons on styrene monomers would be broken and a new σ bond between styrene monomers would be formed, which is substantially stronger than the π bond. Therefore, a long chain of polystyrene would be generated on lignin backbone. As polymerization reaction progressed, the reaction medium became highly heterogeneous, which was different from the uniform suspension system started at the beginning. The heterogeneity could be a sign of producing a more hydrophobic chemical, resulting from grafting polystyrene chains onto lignin backbone. As styrene had only aromatic structure and lacked any functional groups, it would render lignin-*g*-styrene polymer more hydrophobic.

### 3.2. Effect of Polymerization Conditions

#### 3.2.1. Effect of Surfactant Type

Surfactants are one of the vital components in emulsion polymerizations, and their role has been investigated widely [43]. It was claimed that polymerization rate was improved, and overall polymerization activation energy was reduced by adding surfactants to the emulsion reaction systems [44]. Various types of surfactants, such as anionic, cationic, and nonionic types, were previously used in emulsion polymerization of polystyrene latex generation [45,46]. In addition, anionic and nonionic surfactants were reported to be the most effective surfactants widely used in emulsion polymerization systems. It was reported that anionic surfactants provided electrostatic stability to the emulsion systems [47]. Nonionic surfactants mostly provide steric stabilization by preventing particle coagulations [48].

**Figure 1:**
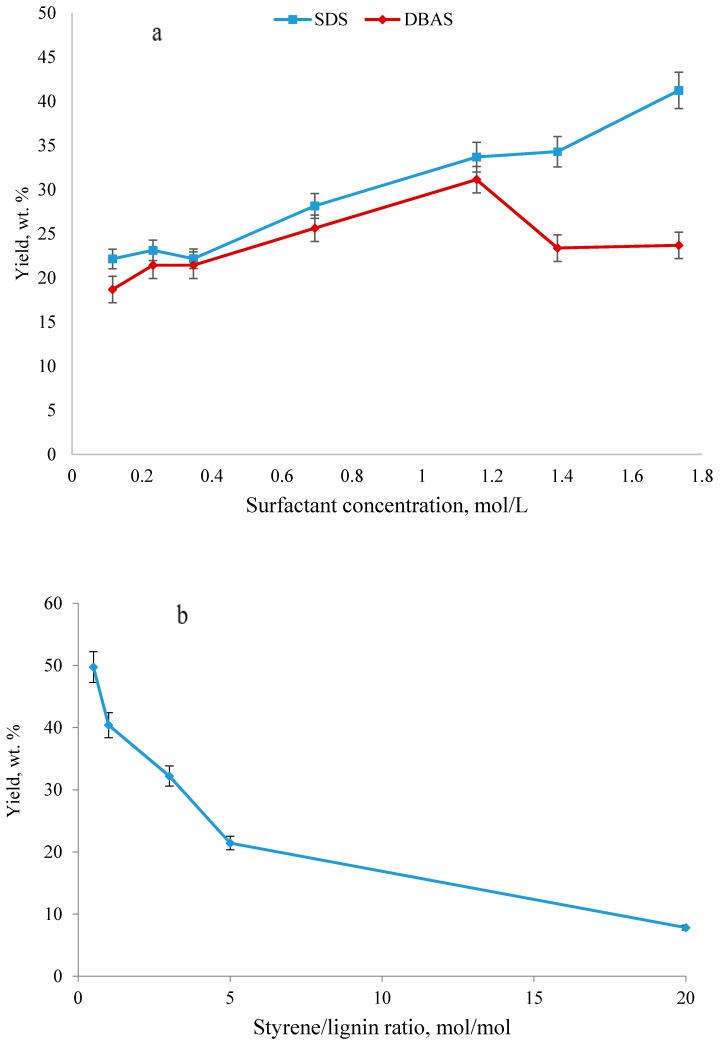

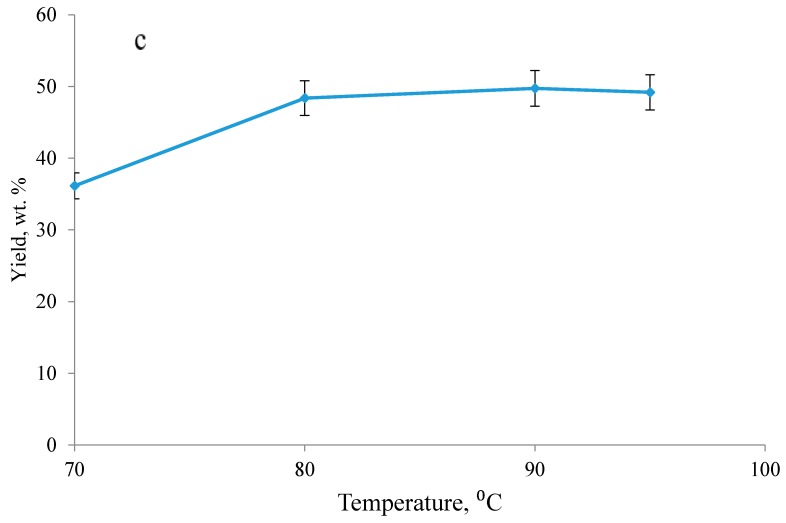
Effect of (**a**) surfactant type and concentration, (**b**) styrene/lignin molar ratio and (**c**) temperature on the yield of polymerization reaction (experiments were performed under the conditions of 100 g/L lignin concentration, pH 1.5 and 2 h). SDS—sodium dodecyl sulfate; DBAS—dodecyl benzenesulfonic acid.

In the present study, the effect of process parameters on the yield of lignin and styrene polymerization was investigated and the results are presented in Figure 1. It is observable that the increase in the dosage of SDS and DBAS improved the yield of polymerization (Figure 1a), while SDS showed slightly better results than DBAS in all dosages. As it was more effective than DBAS, it was selected for further analysis.

#### 3.2.2. Effect of Styrene/Lignin Ratio

In the present work, the lowest styrene/lignin molar ratio with 0.2 mol/L styrene concentration generated the highest yield in the reaction (Figure 1b). The high amount of styrene might have led to the more favorable production of polystyrene. On the other hand, as more styrene molecules were grafted on the lignin radical, viscosity of the suspension would increase; therefore, it is possible that the diffusion of new styrene molecules or polystyrene radicals to the lignin polymer radical would be limited. In other words, propagation on the macroradical sites of lignin-*g*-styrene may not proceed, leading to the higher production of homopolymer. As reported in the past, by increasing the ratio of styrene to chitin, the grafting of styrene on chitin was improved because of more diffusion of styrene in the macroradical sites of chitin [40]. In another report on the graft polymerization of styrene and soy protein, the grafting was improved by increasing styrene concentration from 0.2 to 0.4 mol/L, but further concentrating styrene in the system adversely affected grafting, which was claimed to be related to the viscosity increase of the reaction system that caused the diffusion of styrene to soy proteins radicals more difficult [49]. Therefore, by keeping the styrene concentration at a low amount, the possibility for producing homopolymer of polystyrene could be reduced and higher yield of KL-*g*-PS may be obtained.

#### 3.2.3. Effect of Reaction Temperature

By increasing reaction temperature from 70 to 100 °C, the yield of the reaction was enhanced (Figure 1c). The same phenomenon was observed in Kong and coworkers’ studies in the polymerization of acrylic acid on lignin [13]. It was also postulated on the polymerization of styrene that, by increasing the temperature from 50 to 80 °C, the monomer conversion and polymerization rate were improved dramatically [50]. It is also known that the initiators’ performance is sensitive to temperature, and a higher temperature would generally lead to a faster decomposition rate of initiators leading to faster radical formation, and therefore a higher polymerization rate and yield [51].

#### 3.2.4. Effect of Time

In a series of reactions, the effect of time on the polymerization of lignin and styrene was investigated. The results revealed that the time of reaction (between 1 and 4 h) had a limited effect on the polymerization yield. Therefore, 2 h of reaction that showed slightly better yield than 1 h was chosen for other experiments.

#### 3.2.5. Solvent and Deoxygenation Effects

The use of solvent in altering the yield of reaction was also considered. In a set of experiments, the reactions were conducted in 10/90 (vol/vol) of DMSO/water, 100 g/L lignin concentration, 5 mol/mol styrene/lignin ratio, pH 2.5, 0.35 mol/L SDS, and 90 °C for 2 h, but results showed that the addition of solvent did not improve the yield of reaction. In another set of experiments, the impact of nitrogen on the polymerization of lignin and styrene was studied under the conditions of 100 g/L lignin concentration, 5 mol/mol styrene/lignin ratio, pH 2.5, 0.35 mol/L SDS, and 90 °C for 2 h. The results supported that nitrogen environment marginally affected the yield of polymerization. It was documented that the presence of oxygen in free radical polymerization, including emulsion polymerization, may have detrimental effects on the polymerization rate [52]. Dissolved oxygen is considered as an impurity in reaction systems that consumes radicals [53]. However, Cunningham and coworkers stated that the presence of oxygen only impacted the molecular weight of polystyrene at a high oxygen concentration at the beginning of the reaction. However, similar molecular weights for polystyrene were obtained at the end of the reaction in absence or presence of oxygen (2.65 or 2.12 × 10^6^ g/mol, respectively) [53]. At early stages of the polymerization, an early termination would happen for the growing chains in the presence of oxygen, which would prevent the propagation step to continue, but probably by consuming residue oxygen, the polymerization would continue to increase the molecular weight of polymers at the later stage of polymerization [53].

### 3.3. Sulfonation

**Scheme 2:**
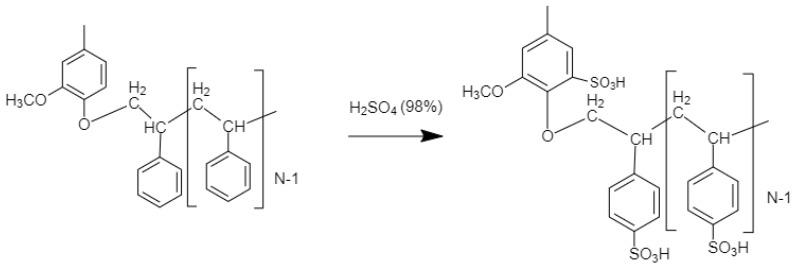
Lignin-styrene polymer sulfonation step.

**Figure 2:**
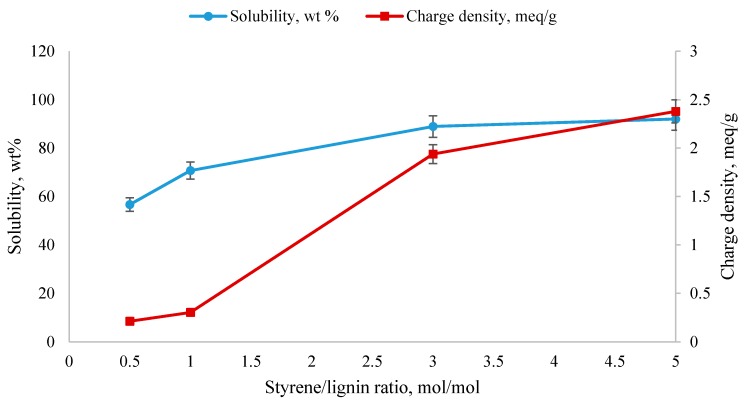
Charge density and solubility of KL-*g*-SPS polymers in 10 g/L water solution (experiments were performed under the conditions of KL-*g*-PS/sulfuric acid 1/10 wt/v and 90 °C for 1 h).

Sulfonation of KL-*g*-PS in concentrated sulfuric acid is a heterogenous reaction. This method of sulfonation has been previously conducted on polystyrene, and partially soluble polystyrene was obtained in a homogenous medium [54]. Scheme 2 shows the sulfonation of KL-*g*-PS. The sulfonate groups could graft on the para position of the aromatic ring of the polymer. Furthermore, steric hinderance is probably the reason for preference of *para* over *ortho* and *meta* positions on the polystyrene chains structure. Sulfonation was conducted on the polymers produced under the conditions of 100 g/L lignin concentration, pH 2.5, 0.35 mol/L SDS, 5 wt % initiator, 90 °C, and 2 h, with different molar ratios of styrene/lignin (0.5, 1, 3, 5). The results for solubility and charge density of different KL-*g*-SPS are shown in Figure 2. It is depicted in Figure 1b that the highest polymerization yield was obtained for the lowest styrene/lignin molar ratio implying more polymerization, and thus presence of styrene on the KL-*g*-PS. The results in Figure 2 show that the more polystyrene chains grafting on lignin, the lower the solubility and charge density obtained for KL-*g*-PSP.

The results in Figure 2 show that the sulfonation of the polymer produced at the lowest styrene/lignin ratio was rather ineffective. An efficient sulfonation of the polymer at the higher styrene/lignin ratio was achieved. As a result, KL-*g*-SPS polymer with the solubility of 92% in deionized water and charge density of −2.4 meq/g in 10 g/L concentration was obtained for the KL-*g*-PS produced at the higher styrene/lignin ratio. Limited sulfonation efficiency with increasing the polymerization yield is probably attributed to the higher molecular weight of the polymers. Linkage between polymer chains was more feasible through cross-linking of polystyrene chains [31]. Steric interruption between long chains of polystyrene might hinder the effectual reaction of sulfuric acid with polystyrene benzene rings on the KL-*g*-PS. Also, the limited sulfonation efficiency may be a result of the chemical nature of the sulfonate group that deactivates the aromatic ring. It was also observed in other studies that polystyrene sulfonation with sulfuric acid resulted in insoluble or partial soluble products in water [29].

### 3.4. Properties of Polymers

#### 3.4.1. NMR Analysis

**Figure 3:**
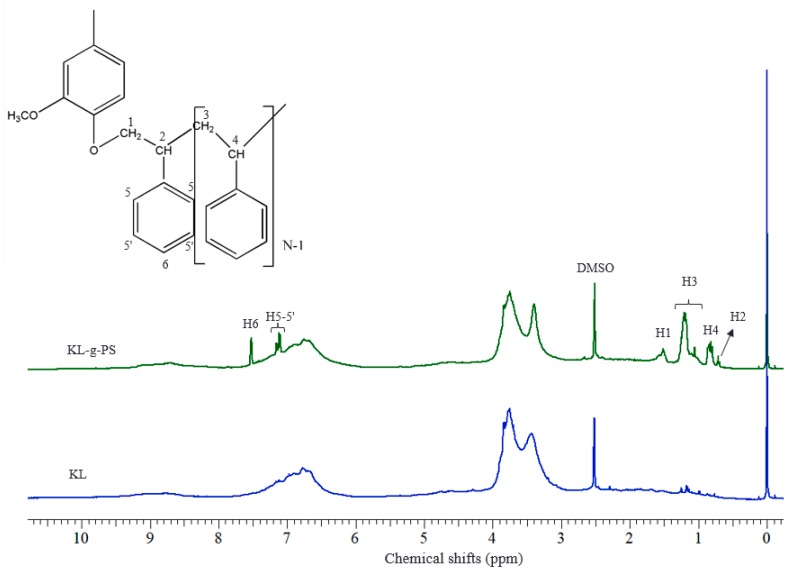
^1^H-nuclear magnetic resonance (NMR) spectrum of KL and KL-*g*-PS in dimethyl sulfoxide (DMSO).

**Figure 4:**
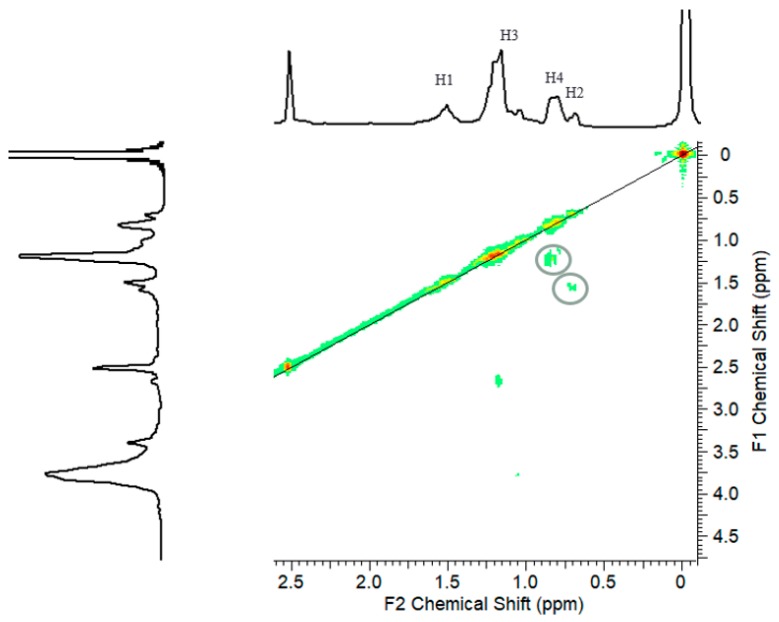
^1^H–^1^H COSY spectrum of KL-*g*-PS in DMSO.

The ^1^H NMR spectra of unmodified lignin and KL-*g*-PS are depicted in Figure 3. The region between 6.0 and 7.5 ppm belongs to aromatic hydrogens of lignin and that between 3.5 and 4.0 ppm belongs to the methoxy group of lignin, which are available in both KL and KL-*g*-PS spectra [55]. The two new signals in the aromatic region in KL-*g*-PS spectrum are attributed to the benzene rings of styrene molecules polymerized on lignin that are shown as H_6_ and H_5-5′_. Polystyrene chain also contains aliphatic parts that contribute to –CH– and –CH_2_ groups [56]. The first molecule of styrene is attached to lignin through formation of ether bond with –CH_2_ group (H_1_). Therefore, a distinguished peak is allocated for the first styrene molecule (H_1_:1.5 ppm and H_2_:0.7 ppm). Other styrene molecules attached to the first styrene molecule are assigned to separate signals at 1.2 and 0.8 ppm relating to –CH_2_(H_3_) and –CH–(H_4_) peaks, respectively. The 2D ^1^H–^1^H COSY was used to confirm the signal assignments in this study. The cross peak duplicated in the Figure 4 confirms that H_2_ signal is coupled by H_1_ signal in the first molecule of styrene. In addition, H_4_ and H_3_ are coupled by each other. Based on NMR results, the grafting degree of 31 mol % of styrene in KL-*g*-PS based on H_3_ signal was obtained.

#### 3.4.2. FTIR Analysis

**Figure 5:**
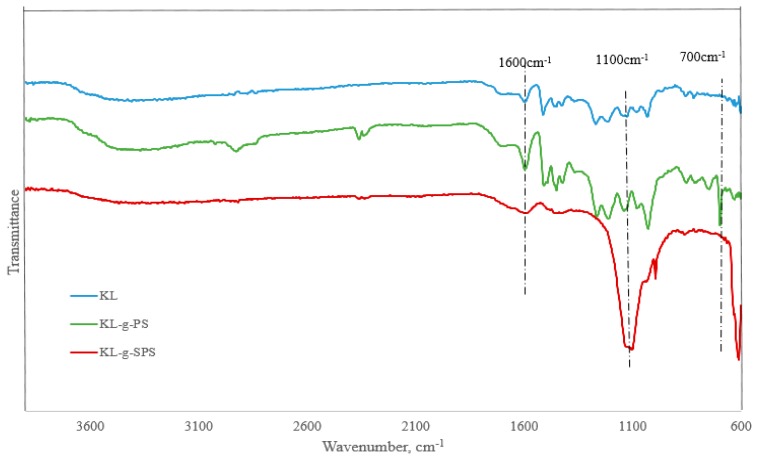
Fourier-transform infrared (FT-IR) spectra of KL, KL-*g*-PS, and KL-*g*-SPS polymers.

The FTIR spectra of KL, KL-*g*-PS, and KL-*g*-SPS are shown in Figure 5. The broad band at 3400 cm^−1^ is assigned to the O–H stretching absorption of the phenolic and aliphatic hydroxyl groups, and a band around 2900 cm^−1^ is assigned to the C–H stretching of the methyl groups [57,58]. Two absorption bands at 1225 and 1140 cm^−1^ are related to the C–O and C–H stretch of guaiacyl unit, respectively, demonstrating that lignin was a softwood species [59]. Peaks at 1601, 1490, and 760 cm^−1^ are related to the CH stretching of aromatic ring, and 1700–2000 cm^−1^ is assigned to the C=C stretching in aromatic ring, which are available on the lignin spectrum as well as on that of other products [60]. A new peak at 700 cm^−1^ appeared on the KL-*g*-PS sample, which was not observed on lignin and is assigned to the C–H stretching of aliphatic C–H (the new σ bond) between the aryl group of polystyrene chain [61]. The same peak also exists in KL-*g*-SPS, but shifted to 615 cm^−1^, probably as a result of sulfonate group addition. In the case of the sulfonated sample (KL-*g*-SPS), the peak at 1100 cm^−1^ is the evidence of S=O bond [22], which is a confirmation for the attachment of sulfonate group to the KL-*g*-PS polymer. 

#### 3.4.3. Molecular Weight and Elemental Analyses 

The carbon, hydrogen, and sulfur contents of KL, KL-*g*-PS, and corresponding sulfonated polymer KL-*g*-SPS are summarized in Table 2. The carbon content of KL-*g*-PS is more, but its oxygen content is less, when compared with KL. Molar ratio of H/C was also calculated based on the data in Table 2. This value is a relative proportion of groups with double bonds, such as cycles and aromatic rings with respect to aliphatic groups [62]. This ratio is 1.05 in KL and 1.1 in KL-*g*-PS, and the increase is attributed to the grafting of styrene monomers on aromatic rings of lignin indicating that the number of double bond equivalents in the KL-*g*-PS polymer was more than double bonds in KL. On the other hand, there is a slight decrease in this value after sulfonation of KL-*g*-PS with sulfuric acid, which can be related to the degradation of some polystyrene chains in a harsh acidic condition. The increase in the sulfur and oxygen contents is indicative of success in sulfonation of KL-*g*-PS and attachment of sulfonate group to the KL-*g*-PS. The structural formula of products based on nine carbons of lignin are shown in Table 2. The C_9_ formula also supports the increment in the sulfur and oxygen contents, but a decrease in the hydrogen content, of lignin via sulfonation and polymerization.

The results of molecular weight analysis are also depicted in Table 2. As expected, the molecular weight of KL-*g*-PS increased markedly with respect to the KL. In contrast, sulfonation with sulfuric acid had a disintegrative effect on the polymer structure, and the final KL-*g*-SPS polymer had the molecular weight of (1.89 ± 0.13) × 10^5^ g/mol, depicting a slight hydrolysis and *MW* reduction under a strong acidic condition.

#### 3.4.4. DSC Analysis

The *T*_g_ analysis was conducted to study the polymer’s molecular mobility and thermal behaviour. The DSC of KL, KL-*g*-PS, and KL-*g*-SPS are tabulated in Table 3. It is seen that KL-*g*-PS had a lower *T*_g_ than KL. The *T*_g_ for polystyrene is reported to be between 90–100 °C [63]. The *T*_g_ of lignin was reported to be in the range of 90–180 °C [64]. The drop in the *T*_g_ was a result of grafting PS on KL. Nonderivatized lignin samples mostly show a high *T*_g_ stemming from the restriction in the thermal mobility of lignin molecules. It is probable that intermolecular hydrogen bonding between hydroxyl functional groups in addition to condensed rigid phenolic moieties would result in thermal behaviour [65]. After polymerization, the elimination of these factors resulted in a *T*_g_ drop. It was reported previously that this phenomenon would happen in miscible polymer blends [66]. The same trend was observed in heat capacity (C_p_) changes of KL and KL-*g*-PS, which would be related to the limited intermolecular interaction after polymerization [67]. After sulfonation, the *T*_g_ of 152 °C was achieved for the KL-*g*-SPS, illustrating a slight increase in *T*_g_. Previous reports on the thermal behaviour of polystyrene after sulfonation displayed the same trend [68]. Polar sulfonate group interaction may be the main reason for this observation, which limits the molecules mobility.

## 4. Conclusions

Sulfonated lignin-*g*-styrene polymer was produced for the first time via emulsion polymerization. The conditions of 100 g/L lignin concentration, pH 2.5, 0.35 mol/L SDS, 5 mol/mol styrene/lignin ratio, 5% initiator, 90 °C, and 2 h generated lignin-*g*-styrene with a 20 wt % yield and grafting degree of 31 mol % based on the quantitative ^1^H-NMR analysis. Molecular weight of KL and KL-*g*-PS were (7.91 ± 0.80) × 10^4^ and (1.05 ± 0.31) × 10^6^ g/mol, respectively. FTIR, NMR, and SLS analyses confirmed the success of the polymerization reaction. Among all factors studied, styrene/lignin molar ratio seemed to have the most significant effect on the yield of reaction, as well as on the sulfonation efficiency. In addition to the factors investigated in this work, other factors, such as pH and initiator dosage, could impact the yield of the polymerization. Therefore, more attempts are required for improving the yield of this polymerization reaction. It was concluded that the higher polystyrene grafting efficiency on lignin led to the less soluble product after sulfonation reaction, and cross-linking of polystyrene chains seems to be the dominant reason for this phenomenon. The sulfonation of the polymer with sulfuric acid generated sulfonated lignin-*g*-styrene polymer with 92% solubility (in a 10 g/L polymer solution) and −2.4 meq/g charge density. The sulfonation caused deduction in molecular weight to (1.89 ± 0.13) × 10^5^ g/mol. The FTIR and elemental analyses confirmed the success of the sulfonation reaction. It is suggested that NMR may be also used for confirming the sulfonation reaction. Regarding the thermal properties of the polymer, limited intermolecular interaction after polymerization probably caused a drop in the *T*_g_ from 156 °C for KL to 149 °C for KL-*g*-PS; however, sulfonation resulted in a slight increment in *T*_g_ to 152 °C for KL-*g*-PSP. Overall, the product was partially soluble in water. The soluble part of the product can be separated and considered as a flocculant for use in water purification, for example, for treating tailing wastes of the mining industry, owing to its high charge density and molecular weight. The insoluble part can also be used as an ion exchange material for water purification purposes. However, further investigations are necessary to unravel the efficiency of these products as flocculants and ion exchange resins.

## Figures and Tables

**Table 1:** Formulation of lignin and styrene emulsion polymerization and corresponding yields (%) of the products.

Exp. No.	SDS (mol/L)	DBAS (mol/L)	Styrene/Lignin (mol/mol)	Temp (°C)	Yield (%) (±2%)
1	0.11	0	5	90	22
2	0.23	0	5	90	23
3	0.35	0	5	90	22
4	0.70	0	5	90	28
5	1.20	0	5	90	34
6	1.40	0	5	90	34.3
7	1.75	0	5	90	41
8	0	0.11	5	90	18
9	0	0.23	5	90	21
10	0	0.35	5	90	21.4
11	0	0.70	5	90	25
12	0	1.20	5	90	31
13	0	1.40	5	90	23.3
14	0	1.75	5	90	23
15	0.35	0	0.5	90	50
16	0.35	0	1	90	40
17	0.35	0	3	90	32
18	0.35	0	5	90	21.4
19	0.35	0	20	90	7.8
20	0.35	0	0.5	70	36
21	0.35	0	0.5	80	48
22	0.35	0	0.5	90	50
23	0.35	0	0.5	100	49

All the experiments were conducted in a 100 g/L lignin concentration, pH 2.5, 5 wt % initiator for 2 h. Sodium dodecyl sulfate (SDS), dodecyl benzenesulfonic acid (DBAS).

**Table 2:** Elemental compositions and molecular weight of KL, KL-*g*-PS, and KL-*g*-SPS.

Lignin Products	Elemental Analysis (wt %)					C_9_ Formula	H/C ^‡^ (mol/mol)	*MW* (g/mol)
	C	H	O ^†^	N	S			
KL	61.6 ± 0.2	5.4 ± 0.1	31.4	0	1.6 ± 0.05	C_9_ H_9.47_ O_3.44_ S_0.08_	1.05	(7.91 ± 0.80) × 10^4^
KL-*g*-PS	65.1 ± 0.3	6.0 ± 0.2	27.3	0	1.6 ± 0.06	C_9_ H_9.95_ O_2.83_ S_0.06_	1.1	(1.05 ± 0.31) × 10^6^
KL-*g*-SPS	38.9 ± 0.2	3.5 ± 0.1	52.2	0	5.4 ± 0.04	C_9_ H_9.72_ O_9.05_ S_1.5_	1.08	(1.89 ± 0.13) × 10^5^

^†^ By difference. ^‡^ The molar ratio of hydrogen and carbon in the sample.

**Table 3:** *T*_g_ and heat capacity of the KL, KL-*g*-S, and KL-*g*-SPS.

Sample	*T*_g_ (°C)	Heat Capacity (C_p_) (J/g °C)
KL	156	0.420
KL-*g*-PS	149	0.075
KL-*g*-SPS	152	0.023

## Date Availability

Raw data of this work is available upon request from the corresponding author.
